# PLM-DBPs: enhancing plant DNA-binding protein prediction by integrating sequence-based and structure-aware protein language models

**DOI:** 10.1093/bib/bbaf245

**Published:** 2025-05-29

**Authors:** Suresh Pokharel, Kepha Barasa, Pawel Pratyush, Dukka B KC

**Affiliations:** Golisano College of Computing and Information Sciences, Rochester Institute of Technology, Rochester 14623, NY, United States; College of Computing, Michigan Technological University, Houghton 49931, MI, United States; Golisano College of Computing and Information Sciences, Rochester Institute of Technology, Rochester 14623, NY, United States; Golisano College of Computing and Information Sciences, Rochester Institute of Technology, Rochester 14623, NY, United States

**Keywords:** plant DNA-binding protein, structure-aware PLMs, protein language models, protein classification, DBP prediction

## Abstract

DNA-binding proteins (DBPs) play a crucial role in gene regulation, development, and environmental responses across plants, animals, and microorganisms. Existing DBP prediction methods are largely limited to sequence information, whether through handcrafted features or sequence-based protein language models (PLMs), overlooking structural cues critical to protein function. In addition, most existing tools are trained for general DBP predictions, which are often not accurate for plant-specific DBPs due to the unique structural and functional properties of plant proteins. Our work introduces PLM-DBPs, a deep learning framework that integrates both sequence-based and structure-aware representations to enhance DBP prediction in plants. We evaluated several state-of-the-art PLMs to extract high-dimensional protein representations and experimented with various fusion strategies to validate the complementary information between the various representations. Our final model, a fusion of sequence-based and structure-aware ANN models, achieves a notable improvement in predicting DBPs in plants outperforming previous state-of-the-art models. Although sequence-based PLMs already demonstrate strong performance in DBP prediction, our findings show that the integration of structural information further enhances predictive accuracy. This underscores the complementary nature of structural representations and establishes PLM-DBPs as a robust tool for advancing plant research and agricultural innovation. The proposed model and other resources are publicly available at https://github.com/suresh-pokharel/PLM-DBPs

## Introduction

DNA-binding refers to the interaction between proteins and DNA, where specific proteins recognize and attach to particular sequences or structures within the DNA molecule in response to cellular signals such as gene expression changes, environmental cues, or molecular interactions. These proteins bind through mechanisms like hydrogen bonding, electrostatic attraction, hydrophobic interactions, and van der Waals forces. Proteins that can bind to DNA are called DNA-binding proteins (DBPs) [1–3]. In humans and other species, this interaction is crucial for various biological processes that include regulating gene expression, DNA replication, repair, and recombination. Understanding the nature of these proteins is crucial for understanding the basis of many diseases and the development of gene-based therapies [1, 4].

Similarly, in plants, DBPs are essential for the regulation of nearly all aspects of plant life, from growth and development to environmental adaptation and defense responses [5]. Since experimental methods are often time-consuming and resource-intensive, numerous computational tools have been developed, with ongoing research focused on advancing these tools to enhance the efficiency and accuracy of their predictions.

Although several computational tools have been developed to predict DBPs in general DBP datasets, there are only a few tools specifically trained to predict DBPs in plants. Plant DBP prediction is crucial not only for basic biological understanding but also has practical applications in agriculture, environmental sustainability, and biodiversity conservation. DBPs in plants differ significantly compared to DBPs in other species and general DNA-binding predictor often struggle to accurately generalize the plant-specific DBPs [5, 6].

Recent advances in computational methods, particularly machine learning and deep learning, have greatly improved the accuracy of DBP (DBP) prediction from protein sequences. However, many of the existing approaches rely on multiple handcrafted features, which require substantial time and effort in the data processing, feature engineering, and model development process. For example, IDRBP-PPCT [7] uses a random forest (RF) model trained on a combined combination of features of position-specific scoring matrix (PSSM), position-specific frequency matrix (PSFM), and cross-transformation (PPCT) features. Similarly, StackDPPred [8] employs a combination of PSSM features, evolutionary distance transformation (EDT) features, residue probing transformation (RPT) features, and a stacked ensemble of support vector machines (SVM), logistic regression, and K-Nearest Neighbor (KNN) models to develop a DBP predictor. These methods incorporate a variety of machine learning techniques, such as SVM, RF, adaptive boosting (ADB), and light gradient boosting (LGB).

Additionally, iDRBP_MMC [9] is a deep learning model that predicts both DBP and RNA-binding protein (RBP) using a multi-label classification approach. It employs PSSM features to train a convolutional neural network (CNN) for predicting four classes: DBPs, RBPs, non-DBPs, and dual RNA–DNA binding proteins (DRBPs). Similarly, DeepDRBP-2L [10] combines a CNN with a bidirectional long short-term memory (biLSTM) network, also trained on PSSM features, to predict the same classes. PDBP-Fusion [11] takes a similar approach by integrating CNN and biLSTM networks, but instead uses one-hot encoded features for training.

Recently, Wu *et al*. [4] proposed two models: a hierarchical CNN-LSTM and a multi-class CNN-LSTM, both trained on PSSM features, achieving balanced prediction accuracy for DBPs and RBPs within a single model. More recently, Qi *et al*. [12] introduced PreDBP-PLMs, a CNN model that combines PLM-based representations with evolutionary (PSSM-based) features to identify general DBPs. Similarly, Zeng *et al*. [13] developed ESM-DBP, which fine-tunes the ESM-2 protein language model (PLM) to create a domain-adaptive language model for DBP prediction.

Despite the development of numerous tools for DBP prediction, only a limited number of these tools are specifically tailored to plant DBPs [5, 6]. To our knowledge, PlDBPred [5] is the most recent tool developed specifically for plant DBP prediction, which is a SVM model trained on the integrated PSSM features. Since existing methods predominantly rely on sequence-based representations and handcrafted features, including PSSM and physicochemical properties, we propose an effective application of both sequence-based and structure-aware PLM-based representations for plant DNA-binding prediction. Sequence-based PLMs are trained on a large corpus of protein sequences that have demonstrated an extraordinary ability to capture meaningful representations of protein sequences. Notable examples include Ankh [14], ESM versions-1/2/3 (evolutionary scale modeling) [15], and ProtT5 [14], which have shown exceptional performance across various protein-related tasks, such as predicting post-translational modifications [16, 17], binding residues in disordered regions [18], and protein structure [19].

On the other hand, with the AlphaFold breakthrough making protein structures more accessible, structure-aware PLMs [20–22] are trained using both sequences as well as 3D structural information. Integrating 3D structural information as a complementary feature enriches protein representations by capturing spatial and functional details not accessible from sequence alone, thereby enhancing predictive performance across a range of tasks.

Building on the success of these sequence-based and structure-aware PLMs, this study explores their potential for predicting DBPs in plants. We investigated several sequence-based PLMs and integrated the optimal model with a structure-aware PLM to develop PLM-DBPs, a robust deep learning framework for plant DBP prediction. Our results demonstrate that combining sequence and structural representations significantly enhances predictive performance, suggesting that such integration captures latent features within proteins that are not apparent from sequence modality alone.

## Materials and methods

### Dataset

The main training and test sets were adopted from PlDBPred [5], which was curated from the UniProt database (accessed on 14 June 2021) [23]. The dataset includes DBPs (positive set, GO: 0003677) and non-DBPs (negative set) derived from plants across 35 species. Sequences containing non-standard amino acids (AA; B, J, O, U, X, Z) and those shorter than 50 AAs were removed. The remaining sequences underwent homology reduction using CD-HIT with a 0.4 sequence identity cutoff. After pre-processing, the final PlDBPred dataset includes 849 DBPs and 1848 non-DBPs. The negative set was balanced by randomly selecting an equal number of non-DBPs. An independent test set with 997 sequences (500 non-DBP and 497 DBPs) is also adopted from PlDBPred. The distribution of DBP and non-DBPs in the training and test sets are presented in Table 1.

**Table 1:** Summary of datasets used in this study

Dataset name	Split/Class	Type	Count
PlDBPred’s Plant Dataset [5]	Train	DBPs	849
		NDBPs	849
	Test-1	DBPs	500
		NDBPs	497
Our Independent Plant Test Set	Test-2	DBPs	20
		NDBPs	20
Multiclass General DBPs Dataset [4]	Train/Val/Test (81:9:10)	SSBs	561
		DSBs	4520
		RBPs	5836
		non-NABPs	12 899

To further assess the generalization capability of the proposed model, an independent test set was curated using a set based on this rule: we selected proteins annotated with the Gene Ontology term GO:0003677 (DNA binding), belonging to the taxonomy group taxonomy ID:33090 (plant species), with reviewed annotations to ensure data quality. We found 20 DBPs which were created between 14 June 2021 and 22 March 2025, to provide an unbiased validation on recent, high-confidence plant protein sequences. The details of the data set provided for this data set are described in Supplementary information (Supplementary Table T2).

Furthermore, to validate our methodology on a more general dataset, we also trained and tested our model using the multiclass DNA-binding dataset developed by Wu and Guo [4]. Details of the dataset and the corresponding results are provided in Supplementary information (Section S2).

### Model workflow architecture

**Figure 1:**
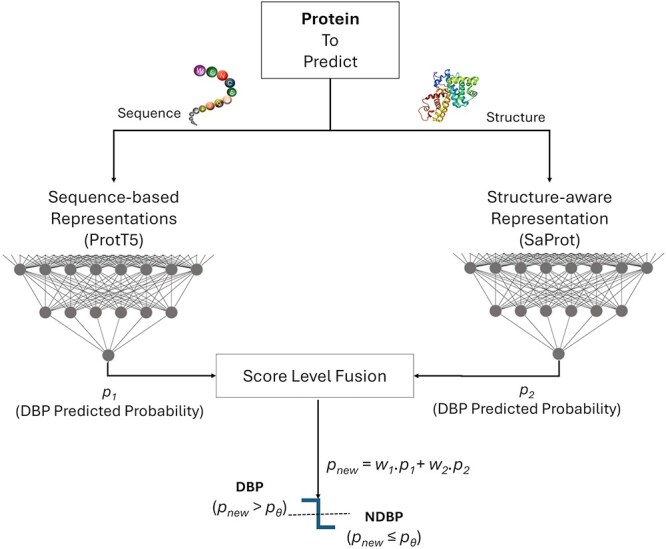
Overview of the proposed workflow for plant DBP prediction, integrating ProtT5 (sequence-based) and SaProt (structure-aware) PLMs. $p_{1}$ and $p_{2}$ represent the probabilities predicted by the two models, which are combined through score-level fusion for more reliable predictions. In this study, equal weights ($w_{1} = w_{2} = 0.5$) are applied, with a cutoff threshold probability of $p_\theta = 0.5$.

An overview of the proposed PLM-DBPs framework is presented in Fig. 1, illustrating the integration of a sequence-based PLM (ProtT5) and a structure-aware PLM (SaProt) for DBP prediction in plants. The AA sequence of a protein is first input into ProtT5, from which an average-pooled, fixed-length protein-level representation of dimension $K \times 1$, where $K = 1024$, is extracted. This representation is then passed through a multi-layer perceptron (MLP) classification head to produce a prediction score.

Concurrently, the corresponding 3Di structural sequence is provided as input to SaProt, which generates residue-level embeddings that are similarly aggregated to form a protein-level representation of dimension $K \times 1$, where $K = 1280$. This structural embedding is also passed through an independent MLP classification head to generate a second prediction score.

The outputs from both MLP heads are fused at the score level using a late fusion strategy—specifically, by computing the weighted average of the individual prediction probabilities. In this study, equal weights are applied to both modalities ($w_{1} = w_{2} = 0.5$), and the final classification is based on a threshold probability of $p_\theta = 0.5$. This fusion strategy leverages the complementary strengths of sequence- and structure-based embeddings, resulting in more robust and accurate DBP predictions. Detailed descriptions of each component depicted in Fig. 1 are provided in the subsequent subsections.

### Protein language models

In the rapidly evolving field of computational biology and bioinformatics, the advent of large PLMs has significantly influenced research and applications across a wide range of bioinformatics tasks. Built on the seminal transformer architecture, these models leverage the attention mechanism to capture long-range dependencies and contextual relationships between AAs within protein sequences. Importantly, these PLMs are trained on extensive corpora of unlabeled protein sequences, enabling them to generate rich representations that encode structural, functional, and evolutionary information. In this study, we utilize several prominent sequence-based PLMs, including Ankh, three versions of ESM-2, and ProtT5. With the widespread availability of high-quality 3D protein structures from AlphaFold, there is a growing interest in bilingual PLMs that are pretrained on both AA sequence and structure data. These models benefit from the complementary information encoded in structural space. To leverage this advantage, we incorporate a leading structure-aware PLM, SaProt, alongside the aforementioned sequence-based models. A comprehensive summary of all the PLMs used in this study is provided in Supplementary information (Section S1). Key details such as model architecture, number of parameters, and training data are also highlighted in Table 2.

**Table 2:** Overview of PLMs evaluated in this study summarizing their architectures, number of layers, number of parameters, training datasets, embedding dimensions, and other details

PLM	Dataset	Architecture	# Layers	# Parameters (Billions)	Embedding Dimension
Ankh-Base	UniRef50	T5 (Hyperparameter Tuning on ProtT5 Framework)	24	$0.45$ B	768
Ankh-Large			24	$1.2$ B	1536
ProtT5	UniRef50	T5-3B	24	$3$ B	1024
ESM2-650M	UniRef50	RoBERTa	33	0.65B	1280
ESM2-3B			36	3B	2560
ESM2-15B			48	15B	5120
SaProt	Sequence, AlphaFold DB, PDB	RoBERTa	33	650M	1280

Ankh, ProtT5 models are taken from Hugging Face (https://huggingface.co) ESM models are downloaded from their GitHub repository (https://github.com/evolutionaryscale/esm) SaProt model is downloaded from their GitHub repository (https://github.com/westlake-repl/SaProt).

#### Extracting protein representation using sequence-based protein language models

**Figure 2:**
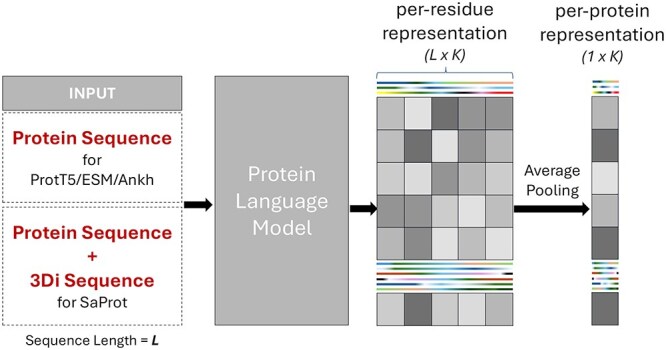
Process of extracting protein representation using sequence-based and structure-aware PLMs. Ankh/ESM-2/ProtT5 takes an AA sequence as input, whereas SaProt takes a combination of the AA sequence and 3Di sequence.

The last hidden state of the encoder component from each sequence-based PLM is utilized as the protein representation for the sequence modality. As illustrated in Fig. 2, a protein sequence of length $L$ is fed to the PLM, resulting in vector embeddings of dimension $K\times 1$ for each AA. Subsequently, these residue-level vector embeddings are consolidated into a protein-level representation using average pooling. For instance, in the case of ProtT5, the model generates a sequence of residue-level embeddings, each of dimension $1024 \times 1$, for a protein sequence of length $L$. These $L$ embeddings are then averaged across the sequence length to produce a single protein-level feature vector of dimension $1024 \times 1$.

#### Extracting protein representation using structure-aware protein language model: SaProt

The 3Di sequence is a structural representation of a protein derived from its 3D conformation. It encodes the local geometric interactions of AAs, allowing the 3D structure of a protein to be expressed in a sequence-like format. As illustrated in Fig. 2, SaProt generates structure-aware embeddings by combining both AA sequences and their corresponding 3D interaction (3Di) sequences.

SaProt takes as input a combined sequence, where each token is formed by pairing a character from the AA sequence with its corresponding 3Di character. It outputs a 1280-dimensional embedding for each token. These token-level embeddings are then average pooled to obtain a fixed-length, protein-level structure-aware representation.

#### Getting 3Di sequence from protein sequence and structure

The concept of 3Di sequence representation is developed by FoldSeek [24] to encode protein structures into sequence-like representations of local conformations, enabling fast structure-based homology searching against large databases.

**Figure 3:**
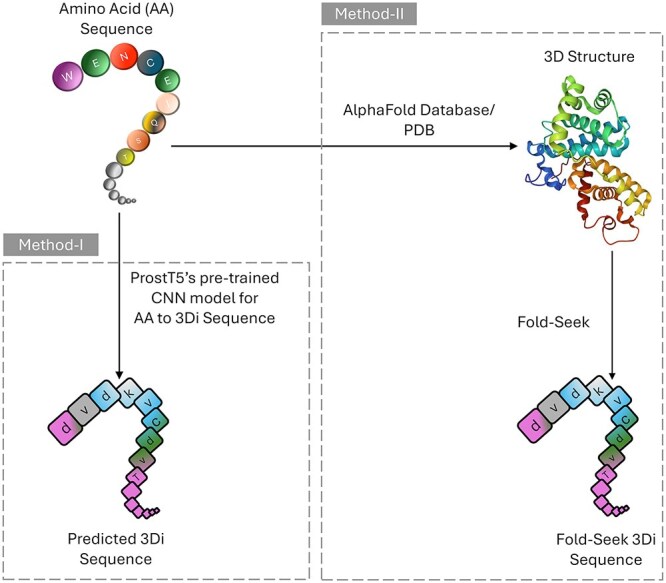
Conversion of AA sequences to 3Di sequences using Method-I: Predictive approach (no 3D structure needed) using the ProstT5’s pre-trained CNN model for 3Di prediction and Method-II: fold-seek (3D structure needed).

As presented in Fig. 3, while Foldseek (Method-II) is the recommended method for deriving 3Di sequences, some protein structures in our dataset were missing from AlphaFold DB or PDB. Due to limited computational resources, we were unable to run AlphaFold locally to obtain the missing 3D structures. Instead, we used ProstT5’s CNN based 3Di [20] prediction model (Method-I) which was trained on millions of AlphaFold-predicted 3D structures and it is helpful for resource-constrained scenarios.

In our small-scale experiments, we compared the performance of SaProt representations generated using Foldseek-derived 3Di sequences versus those generated using ProstT5-predicted 3Di sequences. The difference in the performance of the downstream task was found to be negligible. Given the high-computational cost of generating 3D structures and running Foldseek, we recommend using ProstT5’s 3Di prediction method to use our model.

### Model selection

#### Model evaluation performance metrics

In this study, we used a range of evaluation metrics to assess model performance, consistent with established methods in the field. Metrics such as area under the receiver operating characteristic curve (AUROC), area under the precision-recall curve (AUPR), sensitivity (Sn), specificity (Sp), Matthew’s correlation coefficient (MCC), and accuracy (ACC) were used. Using standard classification terminology, TP (true positive) refers to correctly predicted DNA-binding sequences, FP (false positive) refers to incorrectly predicted DNA-binding sequences, TN (true negative) refers to correctly identified non-DNA-binding sequences, and FN (false negative) refers to misclassified non-DNA-binding sequences.

Sensitivity: $\displaystyle\frac{TP}{TP + FN}$

Specificity: $\displaystyle\frac{TN}{TN + FP}$

Accuracy: $\displaystyle\frac{TP + TN}{TP + FP + TN + FN}$

Precision: $\displaystyle\frac{TP}{TP + FP}$

F1-score: $\displaystyle\frac{2 \cdot \textrm{Precision} \cdot \textrm{Recall}}{\textrm{Precision} + \textrm{Recall}}$

MCC: $\displaystyle\frac{(TP \cdot TN) - (FP \cdot FN)}{\sqrt{(TP + FP)(TP + FN)(TN + FP)(TN + FN)}}$

#### Cross-validation and hyperparameter-tuning

In this study, a five-fold cross-validation is performed to assess the performance of various machine learning models trained on our datasets. The dataset was randomly split into five equal folds, with one-fold serving as the validation set in each iteration, while the remaining four-folds form the training set. The performance on each validation set is presented in Table 4, with final results representing the averages across all folds. To select the best model and hyperparameters, we performed a grid search training over a wide range of configurations for SVM, RF, feed-forward artificial neural network (ANN), and CNN-1D (1D-CNN) models. We used KerasTuner [25] to optimize the hyperparameters of the neural network and CNN-1D models. The search space for the grid search and KerasTuner, as well as the best-performing hyperparameters, are provided in Supplementary information (Section S2). The best-performing model was selected based on its balanced performance across multiple metrics, including MCC, AUROC, and AUPR, obtained through cross-validation.

#### Cross-modality fusion strategies

Based on evaluating individual models using different representations, we explored various fusion strategies to integrate the knowledge of sequence-based and structure-aware representations. We performed five-fold cross-validation of early fusion (feature-level fusion), cross-attention fusion, and late-fusion (score-level fusion) between top-performing sequence-based representation with structure-aware representations. This integrated model leverages the complementary strengths captured from both sequence and structural modalities, allowing them to enhance prediction accuracy mutually.

- **Feature-level fusion (Early fusion):** Combines sequence-based and structure-aware embeddings by concatenating them into a single feature vector before feeding them into the model.
- **Cross-attention fusion:** Applies cross-attention to capture dependencies between sequence-based and structure-aware representations by first projecting both embeddings to a similar dimension latent space. The sequence-based embeddings act as the query $Q$, while the structure-aware embeddings serve as the key $K$ and value $V$. Multihead cross-attention is performed, followed by concatenation of the attended representations, which are then passed through flattened and dense layers for final prediction.
- **Score-level fusion (Late fusion):** Integrates the predicted probabilities from independently trained sequence-based and structure-aware models using a weighted average approach.

#### Final model training

Protein-level representations extracted from both sequence-based and structure-aware PLMs are individually trained using custom prediction heads. Based on the cross-validation results from experiments on various individual and integrated training using representations from sequence-based and structure-aware PLMs, we developed the final model integrating ProtT5 and SaProt-based representations. The final model, dubbed PLM-DBPs (PLMs for DBP prediction), predicts DBPs by averaging the outputs from two independently trained neural networks: one leveraging sequence-based embeddings from ProtT5, and the other utilizing structure-aware embeddings from SaProt. Detailed architectures of these two base models are presented in Table 3. The training statistics of models are provided in Supplementary information (Section S3)

**Table 3:** Detailed model summary of ProtT5-ANN and SaProt-ANN used in the plant DBP prediction workflow

Description	ProtT5-ANN	SaProt-ANN
Input shape	(None, 1024)	(None, 1280)
Hidden layers	4	3
Neurons per layer	512, 512, 128, 16	512, 256, 16
Dropout	0.2–0.4	0.2–0.4
Activation function	ReLU	ReLU
Output layer	1 neuron (Sigmoid)	1 neuron (Sigmoid)
Total parameters	855 201	791 329

**Table 4:** The comparison of five-fold cross-validation results based on embeddings from ProtT5, Ankh, ESM2-650M, ESM2-3B, ESM2-15B, and SaProt

PLM	Model	Sn	Sp	ACC	Pr	F1-score	AUC–ROC	AUC–PR	MCC
ProtT5	SVM	**94.2$\pm $2.51**	91.3$\pm $1.34	**92.8$\pm $1.01**	91.5$\pm $0.82	**92.9$\pm $1.55**	92.8$\pm $2.32	89.1$\pm $1.42	**85.6$\pm $1.12**
	RF	87.9$\pm $2.84	91.1$\pm $1.29	89.5$\pm $1.16	90.8$\pm $1.82	89.3$\pm $2.74	89.5$\pm $0.95	85.8$\pm $1.22	79.0$\pm $1.08
	ANN	93.3$\pm $0.02	91.5$\pm $0.04	92.4$\pm $0.02	91.8$\pm $0.03	92.5$\pm $0.01	**97.3$\pm $0.01**	97.2$\pm $0.01	84.9$\pm $0.03
	CNN1D	92.6$\pm $0.06	91.4$\pm $0.03	92.1$\pm $0.02	91.7$\pm $0.02	92.1$\pm $0.01	96.1$\pm $0.01	96.6$\pm $0.01	84.2$\pm $0.03
ESM2-650M	SVM	92.6$\pm $1.68	92.1$\pm $3.97	92.3$\pm $2.74	92.2$\pm $3.51	92.4$\pm $2.54	92.3$\pm $2.74	89.1$\pm $3.81	84.7$\pm $5.46
	RF	86.1$\pm $2.41	88.1$\pm $5.45	87.2$\pm $3.65	88.2$\pm $4.76	87.1$\pm $3.27	87.1$\pm $3.60	82.9$\pm $4.51	74.3$\pm $7.34
	ANN	92.9$\pm $0.02	90.2$\pm $0.02	91.6$\pm $0.01	90.5$\pm $0.02	91.7$\pm $0.01	96.9$\pm $0.00	97.0$\pm $0.01	83.2$\pm $0.02
	CNN-1D	92.6$\pm $0.02	90.4$\pm $0.04	91.5$\pm $0.02	90.8$\pm $0.02	91.7$\pm $0.02	96.4$\pm $0.01	96.1$\pm $0.02	83.1$\pm $0.04
ESM2-3B	SVM	91.1$\pm $1.85	92.1$\pm $2.25	91.6$\pm $1.23	92.0$\pm $2.50	91.6$\pm $1.51	91.6$\pm $1.22	88.3$\pm $2.54	83.3$\pm $2.47
	RF	83.1$\pm $1.49	91.9$\pm $1.13	87.5$\pm $0.66	91.1$\pm $1.35	86.9$\pm $1.16	87.5$\pm $0.80	84.1$\pm $1.82	75.3$\pm $1.42
	ANN	91.7$\pm $0.02	92.4$\pm $0.02	92.0$\pm $0.01	92.3$\pm $0.02	92.0$\pm $0.02	96.9$\pm $0.01	97.1$\pm $0.01	84.0$\pm $0.01
	CNN-1D	92.8$\pm $0.03	91.9$\pm $0.02	92.1$\pm $0.01	91.8$\pm $0.02	92.1$\pm $0.02	97.2$\pm $0.01	**97.3$\pm $0.02**	83.2$\pm $0.02
ESM2-15B	SVM	92.2$\pm $1.42	**93.4$\pm $2.62**	92.8$\pm $1.62	**93.4$\pm $2.21**	92.8$\pm $1.39	92.8$\pm $1.62	90.0$\pm $2.11	**85.6$\pm $3.25**
	RF	84.3$\pm $3.37	90.8$\pm $4.10	87.6$\pm $2.84	90.5$\pm $3.64	87.2$\pm $2.53	87.5$\pm $2.68	84.1$\pm $3.14	75.4$\pm $5.66
	ANN	90.3$\pm $0.02	92.5$\pm $0.02	91.5$\pm $0.01	92.4$\pm $0.01	91.4$\pm $0.01	96.9$\pm $0.01	96.9$\pm $0.02	82.9$\pm $0.02
	CNN-1D	92.4$\pm $0.03	89.0$\pm $0.03	90.6$\pm $0.01	89.3$\pm $0.02	90.8$\pm $0.02	96.7$\pm $0.01	96.6$\pm $0.02	81.4$\pm $0.02
Ankh	SVM	82.9$\pm $1.59	80.7$\pm $2.01	81.8$\pm $1.06	81.1$\pm $2.44	82.0$\pm $1.29	81.8$\pm $1.06	75.8$\pm $2.24	63.7$\pm $2.12
	RF	77.3$\pm $2.83	77.4$\pm $1.31	77.4$\pm $1.34	77.3$\pm $2.05	77.3$\pm $2.12	77.4$\pm $1.35	71.1$\pm $2.47	54.7$\pm $2.68
	ANN	83.2$\pm $0.02	81.3$\pm $0.01	79.7$\pm $0.03	82.8$\pm $0.01	83.0$\pm $0.02	80.1$\pm $0.02	76.1$\pm $0.02	64.9$\pm $0.03
	CNN-1D	77.6$\pm $0.06	79.9$\pm $0.03	78.8$\pm $0.03	79.5$\pm $0.14	78.5$\pm $0.04	86.8$\pm $0.02	86.4$\pm $0.03	57.7$\pm $0.06
SaProt	SVM	92.5$\pm $2.28	89.6$\pm $1.73	91.6$\pm $1.23	90.9$\pm $1.78	91.7$\pm $1.24	96.6$\pm $0.58	96.9$\pm $0.68	83.2$\pm $2.50
	RF	86.5$\pm $2.19	86.8$\pm $3.11	87.0$\pm $1.57	87.4$\pm $2.19	86.9$\pm $1.58	93.7$\pm $0.89	93.7$\pm $1.24	74.0$\pm $3.14
	ANN	92.8$\pm $3.11	91.6$\pm $1.91	92.2$\pm $1.57	91.6$\pm $1.98	92.1$\pm $1.85	96.4$\pm $1.05	96.5$\pm $0.96	84.4$\pm $3.09
	CNN-1D	90.9$\pm $2.77	90.9$\pm $3.81	90.9$\pm $2.27	90.8$\pm $4.06	90.9$\pm $2.13	96.5$\pm $0.86	96.7$\pm $1.01	81.9$\pm $4.75

Bold values indicate the maximum values for that column. All the metrics are converted to percentages for better comparison. The SaProt results are highlighted to distinguish the outcomes of the structure-aware PLM from those of the sequence-based PLMs.

Fusion strategy: As illustrated in Fig. 1, the score level fusion (also known as late fusion) with a cut-off threshold of $p_\theta = 0.5$ is used to integrate two models described in Table 3. The integrated probability $p$ is calculated as a weighted average of the predicted probabilities from two models, where $p = w_{1} p_{1} + w_{2} p_{2}$. Here, $p_{1}$ and $p_{2}$ represent the predicted probabilities from models that were independently trained using sequence-based (ProtT5) and structure-aware (SaProt) PLMs. In this case, the weights are set as $w_{1} = w_{2} = 0.5$, indicating an average score level fusion approach.

## Results and discussion

### Ablation study on individual PLM representation

The five-fold cross-validation results for hyperparameter optimization and model selection for different prediction heads, including SVM, RF, feed-forward artificial neural networks (ANN), and (1D CNNs) CNN-1D, were trained with a wide range of hyperparameters to evaluate the effectiveness of different feature types.

Table 4 shows that multiple models yield comparable results, indicating high-quality protein representations from both sequence-based and structure-aware PLMs. While other sequence-based PLM’s SVM and CNN-1D models perform similarly to the ANN model, we selected the ProtT5-based ANN model for further experiments due to its superior AUROC and competitive performance across other metrics. In addition, our results are consistent with findings from previous studies [18, 26, 27], which emphasize that the rich information content in PLM-based embeddings allows the downstream model to utilize a relatively simple architecture. We observed that most classification algorithms exhibited consistently strong performance with minimal variation. This enabled us to concentrate on simpler yet effective models, eliminating the need for additional complexity. Consequently, our findings highlight that straightforward ANN models or even shallow architectures can achieve results comparable to deeper neural networks, such as the CNN-1D utilized in our experiments, for downstream tasks.

Similarly, it is noteworthy that models trained with structure-aware protein sequence representations achieve performance nearly equivalent to that of the top-performing models utilizing sequence-based representations.

### Ablation study on sequence-structure representation fusion strategies

**Figure 4:**
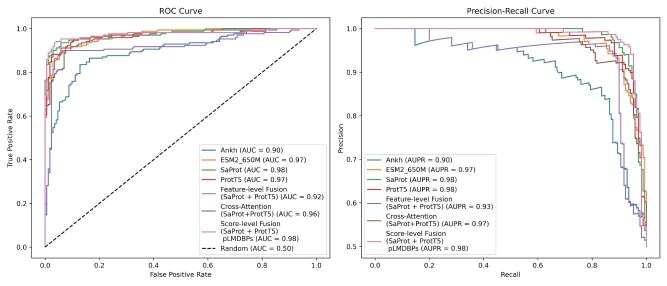
Receiver operating characteristic–area under the curve (ROCAUC) and AUPR comparing the performance of individual PLMs and fusion-based models tested on the first-fold of training set.

Based on the cross-validation results of individually trained models, we explore various model fusion strategies, including feature-level fusion, cross-attention fusion, and score-level fusion, by integrating the best-performing sequence-based and structure-aware PLMs. The results of the five-fold cross-validation, presented in Table 5 and Fig. 4, indicate that score-level fusion gives on-par results or outperforms other strategies across most of the evaluation metrics. In contrast, feature-level fusion and cross-attention fusion did not yield competitive performance, which we attribute to the increased feature dimensionality and model complexity, potentially leading to suboptimal learning and generalization. The

**Table 5:** The comparison of various fusion strategies of ProtT5 (sequence-based) and SaProt (structure-aware) representation

Fusion technique	Sn	Sp	ACC	Pr	F1-score	AUC–ROC	AUC–PR	MCC
Feature-level fusion	**92.6$\pm $0.02**	86.1$\pm $0.04	89.2$\pm $0.02	86.8$\pm $0.04	89.3$\pm $0.02	92.1$\pm $0.03	91.1$\pm $0.02	79.4$\pm $0.03
Cross-attention fusion	89.5$\pm $0.02	93.4$\pm $0.05	91.5$\pm $0.02	93.60$\pm $0.04	91.5$\pm $0.02	97.1$\pm $0.01	96.9$\pm $0.01	83.3$\pm $0.04
Score-level fusion (PLM-DBPs)	92.4$\pm $0.01	**94.2$\pm $0.01**	**93.4$\pm $0.01**	**94.2$\pm $0.01**	**93.4$\pm $0.01**	**98.0$\pm $0.01**	**97.9$\pm $0.01**	**86.8$\pm $0.02**

Bold values indicate the maximum values for that column.

### Analysis of features

We used UMAP (uniform manifold approximation and projection) [28] to visualize high-dimensional representations in a lower-dimensional space. Specifically, we generated 2D UMAP projections for both the original ProtT5 and SaProt embeddings. We also visualized their refined representations learned by the respective PLM-DBPs base models, illustrating the distinction between DBPs and non-DBPs.

**Figure 5:**
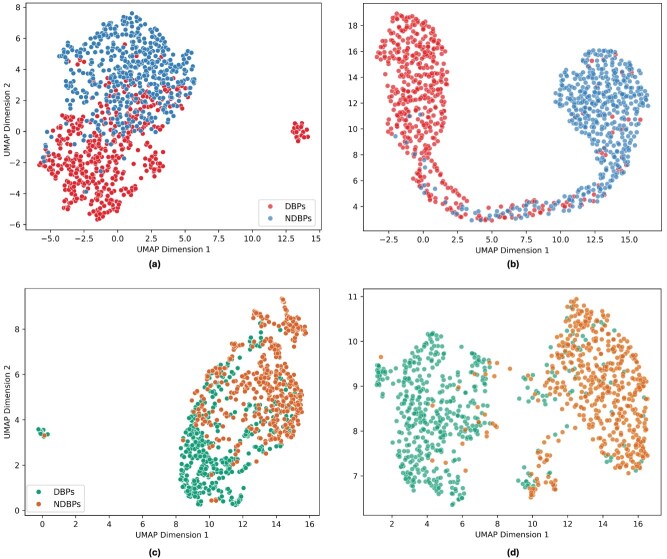
Visualization of 2D UMAP projections of learned feature representations. (a) Raw ProtT5 embeddings, (b) ProtT5-based ANN model embeddings from the penultimate layer, (c) raw SaProt embeddings, and (d) SaProt-based ANN model embeddings from the penultimate layer.

As illustrated in Fig. 5a and c, the raw ProtT5 and SaProt embeddings exhibit noticeable separation between DBP and NDBP proteins in our experiment. However, after training the PLM-DBPs models using these features, the resulting embeddings (Fig. 5b and d) demonstrate even clearer distinctions. While these visualizations are lower-dimensional approximations, the observed separations highlight the effectiveness of our proposed approach in capturing the hidden patterns in DBP and non-DBPNDBP.

### Comparison with existing DNA-binding prediction tools

As presented in Table 6, we compared the results of our proposed model with existing tools DNAbinder [29], DPP_PseAAC [30], IDRBP_MMC [9], IDRBP-PPCT [7], PDBP-fusion [11], DeepDRBP [10], StackDPPred [8], PlDBPred[5], and ESM-DBP [13] on the independent test set described in Table 6. The published results from PlDBPred [5] are included for comparison, as it employs the same training and test datasets as our study.

**Table 6:** Comparison of performance between PLM-DBPs and existing tools on independent test set

Method	Sn	Sp	ACC	Pre	F1-score	MCC
DNAbinder	50.9	51.5	51.0	67.4	57.9	2.1
DPP_PseAAC	56.1	54.6	55.4	49.8	52.8	10.9
IDRBP_MMC	72.7	67.7	69.9	63.8	67.9	40.1
IDRBP-PPCT	63.9	84.9	69.9	91.4	75.2	44.1
PDBP-fusion	86.4	70.0	75.8	61.2	71.7	54.0
DeepDRBP	61.4	70.6	64.7	79.0	69.1	30.7
StackDPPred	65.8	61.1	63.0	54.2	59.4	26.4
PlDBPred	82.6	93.4	88.0	92.6	87.3	76.4
ESM-DBP^a^	**95.5**	87.1	90.69	84.5	89.71	81.7
PLM-DBPs^a^	88.2	95.4	91.9	94.6	91.3	84.0
PLM-DBPs	89.8	**95.1**	**92.3**	**95.4**	**92.5**	**84.7**

^a^For a fair comparison, we removed 41 positive sequences from our test dataset when comparing with ESM-DBP, as they were part of its training set (UniDB40). Bold values indicate the maximum values for that column.

Our comparative analysis evaluated the proposed model against existing approaches using several key metrics: sensitivity, specificity, accuracy, precision, F1-Score, and MCC. Although AUROC and AUPR are not included in Table 6 as some of the existing tools that we are comparing against have not reported these metrics. However, PLM-DBPs achieved impressive AUROC and AUPR values of 97.5 and 97.3, respectively on the independent test set.

Additionally, to further evaluate the effectiveness of our proposed method, we conducted experiments on a multiclass general DBP dataset recently analyzed by Wu and Guo [4]. This dataset comprises four distinct classes: RBP, single-stranded DBPs (SSB), double-stranded DBPs (DSB), and non-nucleic acid-binding proteins (NABP). The results of this evaluation are presented in Supplementary information (Section S2), which demonstrates that our method performs well in general DBP prediction, effectively distinguishing between related protein classes.

### Comparison with the plant specific DNA-binding prediction tool (PlDBPred)

For further validation of our proposed model, we curated a new test set with the following criteria: proteins associated with the go_manual:0003677 term (DNA binding), from the taxonomy group taxonomy_id:33090 (representing plant species), with reviewed annotations (ensuring high-quality data), and created between 14 June 2021 and 22 March 2025. For the negative class (non-DBPs), we followed the same criteria but excluded proteins associated with go_manual:0003677. This resulted in 951 entries, from which we selected the first 20 sequences for use in our validation.

This ensures that all proteins in this test set are created after our training and main test dataset, preventing any overlap. This independent dataset was specifically designed to assess the generalization and robustness of our model on recent, high-quality protein data, ensuring an unbiased evaluation. Our model achieved competitive performance on this dataset, with an MCC of 85.9%. Detailed results of this experiment are provided in Supplementary information, Supplementary Table III.

We compared the performance of PLM-DBPs against PlDBPred (state of the art model for plant-specific DNA-binding) and found that our model identifies two additional DBPs with high confidence in nearly all cases, outperforming PlDBPred’s results. Detailed results are presented in Supplementary information (Supplementary Table T4).

### Computational efficiency and runtime analysis of model inference

**Figure 6:**
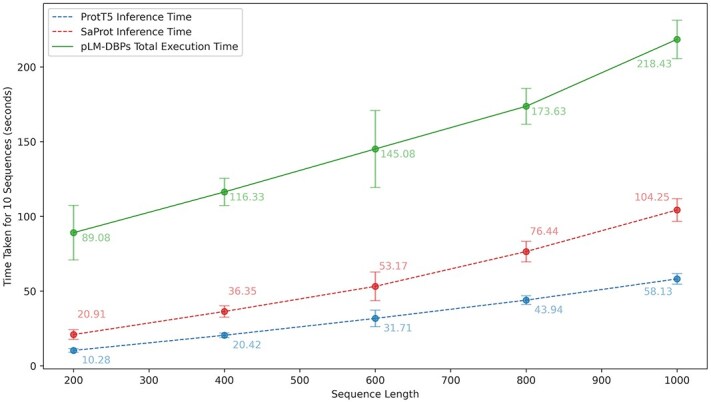
PLM-DBPs model inference time analysis for predicting protein sequences of varying lengths using Intel$\circledR $ Xeon$\circledR $ (8 cores, 16 threads) @ 3.70 GHz with 32 GB DDR4 RAM.

For our experiments, we utilized a single A100 80GB GPU for model training. However, inference and evaluation were conducted on an Intel$\circledR $ Xeon$\circledR $ W-2145 processor (8 cores, 16 threads) @ 3.70GHz with 32GB DDR4 ECC RAM (2 $\times $ 16 GB, 2666 MHz, Samsung). We observed that prediction time increases with sequence length. To systematically estimate computation time, we conducted experiments on 1000 randomly generated protein sequences of varying lengths (200–1000, in intervals of 200). Figure 6 illustrates the time required for generating representations and the overall prediction time using our proposed tool. On average, the time taken to predict 10 sequences of lengths 200, 400, 600, 800, and 1000 was approximately 89 $\pm \ $18, 116 $\pm \ $9, 145 $\pm \ $25, 173 $\pm \ $12, and 218$\ \pm \ $13 s, respectively. Further details of computational time analysis are provided in Supplementary information (Supplementary Table T1). The prediction time can be significantly reduced by using a GPU to generate representations from PLMs.

### Conclusion and future work

In this study, we introduced PLM-DBPs, a robust prediction model specifically designed for DBPs in plants, integrating sequence-based and structure-aware PLM representations. After exploring various state-of-the-art PLMs for protein representation, we developed an integrated model leveraging a score-level fusion of models trained using ProtT5 (sequence-based PLM) and SaProt (structure-aware PLM) representations.

Our final model demonstrated a notable improvement in most of the measured metrics that highlight the complementary benefits of sequence-based and structure-aware PLMs. By moving beyond reliance on purely sequence-based handcrafted features or embeddings, our proposed methodology integrates structural information, leading to more accurate identification of DBPs in plants. Further validation was conducted through the curation of an additional independent test set comprising plant DBPs annotated after our original dataset’s compilation. The results in all experimented datasets demonstrated that PLM-DBPs have better generalization and robustness as compared to existing approaches.

Future work will involve benchmarking advanced sequence-based PLMs such as ESM3 [31], xTrimoPGLM [32] and explore PLMs more to further enhance prediction accuracy. Additionally, we plan to explore other more efficient fusion techniques and weighting strategies to optimally integrate sequence-based and structure-aware PLMs, e.g. dynamically assigning their weights based on their contributions in making decisions (currently, as illustrated in Fig. 1, we assign equal weights $(w_{1} = w_{2} = 0.5)$ to each prediction). Furthermore, we aim to explore other structure-aware models and incorporate diverse structural databases to improve the model’s generalizability and robustness. A key direction will be to explore the techniques to incorporate structural information more effectively, as the current approach leverages only limited structural knowledge through 3Di sequences.

**Key Points**

- PLM-DBPs is a novel method that predicts plant DNA-binding proteins (DBPs) by integrating representations from both sequence-based and structure-aware protein language models (PLMs).
- We systematically evaluated five different sequence-based PLMs and combined the best-performing one (ProtT5) with a structure-aware model (SaProt), exploring multiple integration strategies, including score-level, feature-level, and cross-attention fusion.
- PLM-DBPs is validated using two independent plant-based test sets, including newly curated data from UniProt, and further assessed its generalizability using a multiclass general DBP dataset.
- PLM-DBPs that use cross-modality fusion consistently outperformed existing plant-specific and general DBP predictors in predicting plant DBPs, achieving superior performance across key evaluation metrics.

## Supplementary Material

suppl_materials_bbaf245

## Data Availability

The stand-alone version of the model is publicly available at https://github.com/suresh-pokharel/PLM-DBPs.
