# Supplementary material for: PLM-DBPs: enhancing plant DNA-binding protein prediction by integrating sequence-based and structure-aware protein language models
Source: Brief Bioinform. 2025 May 29;26(3):bbaf245. doi: 10.1093/bib/bbaf245 (PMC12121366; doi:10.1093/bib/bbaf245)
Supplement: suppl_materials_bbaf245 [file suppl_materials_bbaf245.docx]

# PLM-DBPs: Enhancing Plant DNA-Binding Protein Prediction by Integrating Sequence-Based and Structure-Aware Protein Language Models

Suresh Pokharel^1^, Kepha Barasa^2^, Pawel Pratyush^1^, and Dukka KC^1^

^1^Golisano College of Computing and Information Sciences, Rochester Institute of Technology, Rochester, NY, 14623, USA

^2^College of Computing, Michigan Technological University, Houghton, MI, 49931, USA

**SUPPLEMENTARY INFORMATION**

# Section S1: Further Description of Protein Language Models

The protein language models (PLMs) used in this study differ in terms of their underlying architectures, parameter sizes, tokenization strategies, and training objectives, all of which influence their ability to capture biologically meaningful representations.

**Ankh**

Ankh is a general-purpose protein language model with a relatively smaller number of parameters compared to other competitive models. It was trained on the UniRef50 dataset, which contains 45 million protein sequences. There are two versions available: Ankh_base and Ankh_large, with 450 million and 1.15 billion parameters, respectively. These output embedding dimensions of 768 and 1536 per amino acid residue. In this study, we used the Ankh_large version, referred to simply as Ankh.

**ESM (Evolutionary Scale Modeling)**

ESM is a BERT-based protein language model trained on the UniRef50 dataset. It predicts masked amino acids using the full sequence context. In this study, we explored three variants of the ESM2 model: ESM2-650M, ESM2-3B, and ESM2-15B, corresponding to esm2_t33_650M_UR50D, esm2_t36_3B_UR50D, and esm2_t48_15B_UR50D.

**ProtT5**

ProtT5-XL-U50, referred to as ProtT5 in this paper, is a protein language model pre-trained on the UniRef50 database using a masked language modeling approach. Based on Google's T5-3B architecture, it has 3 billion trainable parameters and 24 layers in both its encoder and decoder. Unlike the original T5, it adopts a BART-like objective instead of span denoising. In this study, only the encoder was used for feature extraction.

**SaProt**

SaProt is trained on approximately 40 million protein sequences along with their AlphaFold2-predicted 3D structures. It uses Foldseek to generate 3Di tokens representing structural information, which are combined with residue tokens to create structure-aware embeddings. Based on the ESM-2 architecture with an expanded embedding layer, SaProt is trained using a masked language modeling objective, masking 15% of structure-aware tokens per batch. It effectively integrates sequence and structure information, making it suitable for tasks such as secondary structure prediction, cleavage site prediction, and protein-protein interaction prediction.

# Section S2: Grid Search and Keras-Tuner Search Space and Hyperparameters

**Keras Tuner Configuration:**

For ANN, Keras Tuner was used with the Random Search strategy to optimize the model. The search space included the number of layers (1–5), units per layer (chosen from 8, 16, 32, 64, 128, 256, 512), dropout rate (0.2, 0.3, 0.4, 0.5), learning rate (log-scaled between 1e-7 and 1e-3), and L2 regularization rate (log-scaled between 1e-7 and 1e-3). The tuner performed 17 trials with 5 executions per trial, optimizing for validation accuracy. The model architecture was constructed using a modular function that incorporated the selected hyperparameters. For CNN-1D, the model was optimized manually observing various combinations of the number of layers, filter sizes, dense layers, dropouts, and other tunable parameters.

**CNN Model (SaProt)**

_________________________________________________________________

Layer (type) Output Shape Param #

==============================================================

input_1 (InputLayer) [(None, 1280, 1)] 0

conv1d (Conv1D) (None, 1276, 64) 384

dropout (Dropout) (None, 1276, 64) 0

conv1d_1 (Conv1D) (None, 1274, 128) 24704

dropout_1 (Dropout) (None, 1274, 128) 0

flatten (Flatten) (None, 163072) 0

dense (Dense) (None, 128) 20873544

dropout_2 (Dropout) (None, 128) 0

dense_1 (Dense) (None, 1) 129

==============================================================

Total params: 20,898,761

Trainable params: 20,898,761

Non-trainable params: 0

_________________________________________________________________

**CNN Model (ProtT5)**

_________________________________________________________________

Layer (type) Output Shape Param #

==============================================================

input_1 (InputLayer) [(None, 1024, 1)] 0

conv1d (Conv1D) (None, 1020, 64) 384

dropout (Dropout) (None, 1020, 64) 0

conv1d_1 (Conv1D) (None, 1016, 128) 41088

dropout_1 (Dropout) (None, 1016, 128) 0

conv1d_2 (Conv1D) (None, 1014, 128) 49280

dropout_2 (Dropout) (None, 1014, 128) 0

conv1d_3 (Conv1D) (None, 1012, 16) 6160

dropout_3 (Dropout) (None, 1012, 16) 0

flatten (Flatten) (None, 16192) 0

dense (Dense) (None, 1) 16193

==============================================================

Total params: 113,105

Trainable params: 113,105

Non-trainable params: 0

**Random Forest Hyperparameters - Grid Search Configurations**

Base Model: RandomForestClassifier(random_state=42)

n_estimators: 10, 50, 100, 150, 200

max_features: 'sqrt', 'log2', None

max_depth: None, 2, 5, 10, 15, 20

criterion: 'gini', 'entropy', 'log_loss'

**Support Vector Machine (SVM) Hyperparameters- Grid Search Configurations**

Base Model: svm.SVC(random_state=42)

C: 0.0001, 0.001, 0.01, 0.1, 1, 10, 100

kernel: 'linear', 'rbf', 'poly'

gamma: 'scale', 'auto', 0.0001, 0.001, 0.9

degree: 1, 3, 6, 9, 12

For ProtT5:

"SVM": svm.SVC(C=1, kernel='linear',gamma=0.0001),

"Random Forest": RandomForestClassifier(n_estimators=150, random_state=40, max_features='sqrt')

For SaProt:

"SVM": svm.SVC(C=2 kernel='linear',gamma=0.001),

"Random Forest": RandomForestClassifier(n_estimators=200, random_state=40, max_features='sqrt')

#

#

# Table T1: Execution Time of PLM-DBPs Models to Predict Ten Randomly Generated Sequences

| **Sequence Length** | **Prott5_time (Seconds)** | **Saprot_time (Seconds)** | **Total execution time - PLMDBPs)**  **(Seconds)** |
| --- | --- | --- | --- |
| 200 | 10.28 ± 1.22 | 20.91 ± 3.24 | 89.08 ± 18.22 |
| 400 | 20.42 ± 1.57 | 36.35 ± 3.82 | 116.33 ± 9.09 |
| 600 | 31.71 ± 5.49 | 53.17 ± 9.57 | 145.08 ± 25.72 |
| 800 | 43.94 ± 2.91 | 76.44 ± 6.89 | 173.63 ± 11.96 |
| 1000 | 58.13 ± 3.54 | 104.25 ± 7.53 | 218.43 ± 12.85 |

#
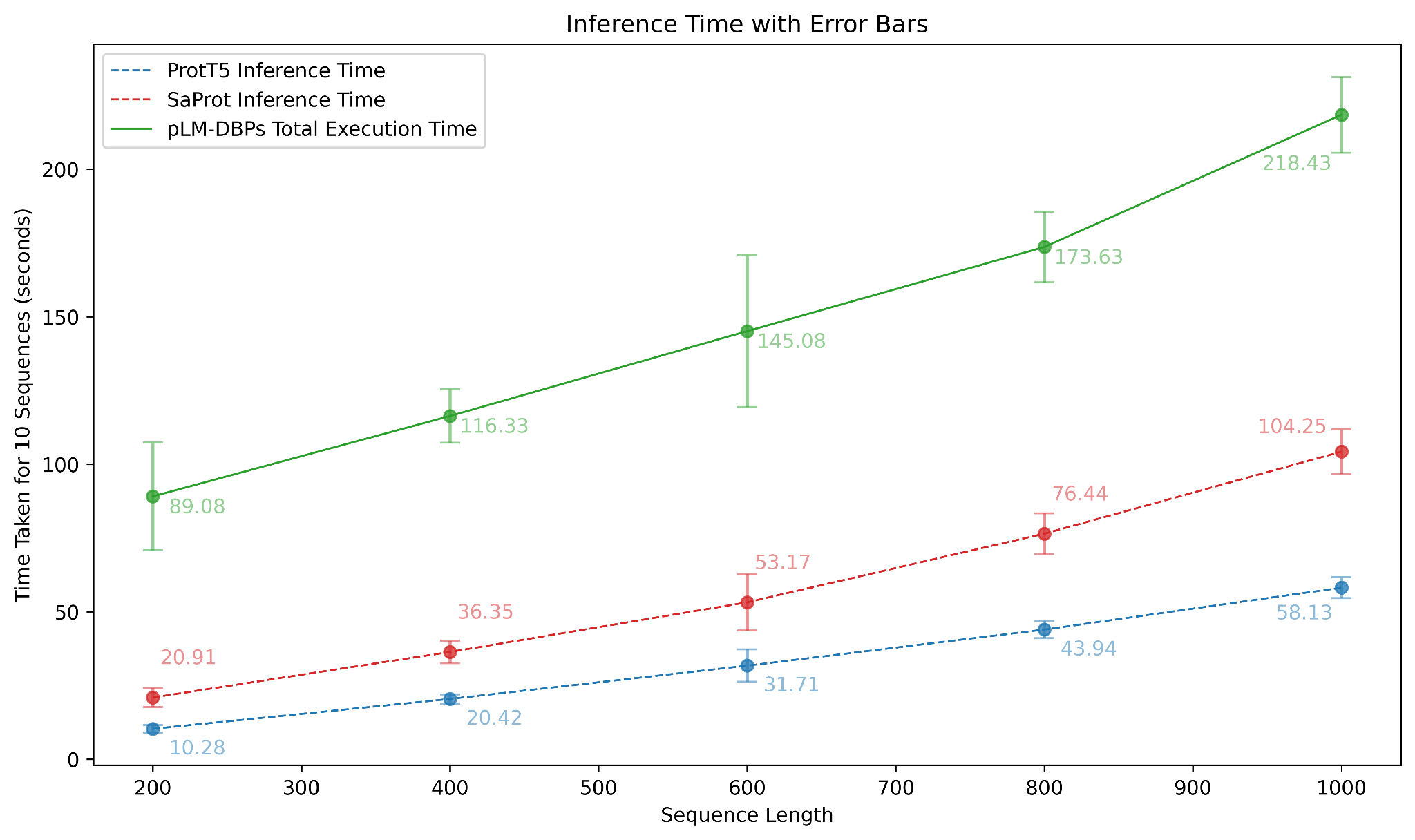


# Table T2: Distribution of Plant DNA-binding proteins in our independent test sets, curated from UniProt (2021–2025)

| **POSITIVE** | | | **NEGATIVE** | | |
| --- | --- | --- | --- | --- | --- |
| **Uniprot ID** | **Organism** | **Label** | **Uniprot ID** | **Organism** | **Label** |
| B6T5Z6 | Zea mays | 1 | A0A067XR63 | Diospyros kaki | 0 |
| F4HZD1 | Arabidopsis thaliana | 1 | A0A067XRK9 | Diospyros kaki | 0 |
| F4I240 | Arabidopsis thaliana | 1 | A0A067YMX8 | Diospyros kaki | 0 |
| I1GN76 | Brachypodium distachyon | 1 | A0A072ULZ1 | Medicago truncatula | 0 |
| K7TLS0 | Zea mays | 1 | A0A072VDF2 | Medicago truncatula | 0 |
| Q0J995 | Oryza sativa subsp. japonica | 1 | A0A075D5I4 | Rauvolfia serpentina | 0 |
| Q4G2I2 | Sorghum bicolor | 1 | A0A075D657 | Vinca minor | 0 |
| Q5W6D6 | Oryza sativa subsp. japonica | 1 | A0A075QQ08 | Nicotiana tabacum | 0 |
| Q652K4 | Oryza sativa subsp. japonica | 1 | A0A076FFM5 | Ocimum basilicum | 0 |
| Q67ZB6 | Arabidopsis thaliana | 1 | A0A0A0LLY1 | Cucumis sativus | 0 |
| Q8H1S7 | Arabidopsis thaliana | 1 | A0A0B6CGH9 | Ocimum basilicum | 0 |
| Q9LN09 | Arabidopsis thaliana | 1 | A0A0E3T3B5 | Malus domestica | 0 |
| B4FNX4 | Zea mays | 1 | A0A0E3T552 | Malus domestica | 0 |
| C0SV12 | Arabidopsis thaliana | 1 | A0A0F7G352 | Vanilla planifolia | 0 |
| K7UPS5 | Zea mays | 1 | A0A0K0PU92 | Lupinus angustifolius | 0 |
| A0A1U8IPT1 | Gossypium hirsutum | 1 | A0A0M3Q1Q3 | Thymus vulgaris | 0 |
| A0A3Q7EKL1 | Solanum lycopersicum | 1 | A0A166U5H3 | Kitagawia praeruptora | 0 |
| B7F9I5 | Oryza sativa subsp. japonica | 1 | A0A178WF56 | Arabidopsis thaliana | 0 |
| Q6F368 | Oryza sativa subsp. japonica | 1 | A0A1D6E0S8 | Zea mays | 0 |
| Q8L7T9 | Arabidopsis thaliana | 1 | A0A1D8EJF9 | Solanum pimpinellifolium | 0 |

**Search Query for Positive:** *(go_manual:0003677) AND (taxonomy_id:33090) AND (reviewed:true) AND (date_created:[2021-06-14 TO 2025-03-22])*

**Search Query for Negative:***NOT (go_exp:0003677) AND (taxonomy_id:33090) AND (reviewed:true) AND (date_created:[2021-06-14 TO 2025-03-22]) Selected first 20 records*

*Note: Fasta files can be found in the GitHub repository.*

# Table T3: Our Model’s Prediction on Independent Test Set Presented in Table T2

| **Uniprot ID** | **Label** | **PLM-DBPs**  **(Probability)** | **y_pred** |  | **Uniprot ID** | **Label** | **PLM-DBPs**  **(Probability)** | **y_pred** |
| --- | --- | --- | --- | --- | --- | --- | --- | --- |
| B6T5Z6 | 1 | 0.9888 | 1 |  | A0A067XR63 | 0 | 0.015 | 0 |
| F4HZD1 | 1 | 0.9098 | 1 |  | A0A067XRK9 | 0 | 0.0127 | 0 |
| F4I240 | 1 | 0.6104 | 1 |  | A0A067YMX8 | 0 | 0.0133 | 0 |
| I1GN76 | 1 | 0.9437 | 1 |  | A0A072ULZ1 | 0 | 0.0446 | 0 |
| K7TLS0 | 1 | 0.9926 | 1 |  | A0A072VDF2 | 0 | 0.0107 | 0 |
| Q0J995 | 1 | 0.9964 | 1 |  | A0A075D5I4 | 0 | 0.0277 | 0 |
| Q4G2I2 | 1 | 0.9902 | 1 |  | A0A075D657 | 0 | 0.0263 | 0 |
| Q5W6D6 | 1 | 0.9978 | 1 |  | A0A075QQ08 | 0 | 0.2639 | 0 |
| Q652K4 | 1 | 0.9953 | 1 |  | A0A076FFM5 | 0 | 0.0325 | 0 |
| Q67ZB6 | 1 | 0.0644 | 0 |  | A0A0A0LLY1 | 0 | 0.3587 | 0 |
| Q8H1S7 | 1 | 0.9289 | 1 |  | A0A0B6CGH9 | 0 | 0.0112 | 0 |
| Q9LN09 | 1 | 0.9847 | 1 |  | A0A0E3T3B5 | 0 | 0.0264 | 0 |
| B4FNX4 | 1 | 0.9872 | 1 |  | A0A0E3T552 | 0 | 0.0252 | 0 |
| C0SV12 | 1 | 0.8652 | 1 |  | A0A0F7G352 | 0 | 0.0109 | 0 |
| K7UPS5 | 1 | 0.9865 | 1 |  | A0A0K0PU92 | 0 | 0.1464 | 0 |
| A0A1U8IPT1 | 1 | 0.9687 | 1 |  | A0A0M3Q1Q3 | 0 | 0.0104 | 0 |
| A0A3Q7EKL1 | 1 | 0.9663 | 1 |  | A0A166U5H3 | 0 | 0.0105 | 0 |
| B7F9I5 | 1 | 0.2117 | 0 |  | A0A178WF56 | 0 | 0.0332 | 0 |
| Q6F368 | 1 | 0.2899 | 0 |  | A0A1D6E0S8 | 0 | 0.0273 | 0 |
| Q8L7T9 | 1 | 0.9795 | 1 |  | A0A1D8EJF9 | 0 | 0.015 | 0 |

**Results:**

TN = 20

FN = 3

FP = 0

TP = 17

Accuracy: 0.925. Precision: 1. Recall: 0.85. F1 Score: 0.919. MCC: 0.8597

# Table T4: Our Model’s Prediction on Independent Test Set (Positive DBPs) Presented in Table T2 with PlDBPred

| **Uniprot ID** | **Label** | **PlDBPred** | | **PLM-DBPs (Our Model)** | |
| --- | --- | --- | --- | --- | --- |
|  |  | **prob** | **y_pred** | **prob** | **y_pred** |
| B6T5Z6 | 1 | 0.99 | 1 | 0.9888 | 1 |
| F4HZD1 | 1 | 0.94 | 1 | 0.9098 | 1 |
| F4I240 | 1 | 0.58 | 1 | 0.6104 | 1 |
| I1GN76 | 1 | 0.82 | 1 | 0.9437 | 1 |
| K7TLS0 | 1 | 0.97 | 1 | 0.9926 | 1 |
| Q0J995 | 1 | 0.98 | 1 | 0.9964 | 1 |
| Q4G2I2 | 1 | 0.96 | 1 | 0.9902 | 1 |
| Q5W6D6 | 1 | 0.59 | 1 | 0.9978 | 1 |
| Q652K4 | 1 | 0.97 | 1 | 0.9953 | 1 |
| Q67ZB6 | 1 | 0.0900 | 0 | 0.0644 | 0 |
| Q8H1S7 | 1 | 0.9200 | 1 | 0.9289 | 1 |
| Q9LN09 | 1 | 0.9500 | 1 | 0.9847 | 1 |
| B4FNX4 | 1 | 0.9600 | 1 | 0.9872 | 1 |
| C0SV12 | 1 | 0.8600 | 1 | 0.8652 | 1 |
| K7UPS5 | 1 | 0.9100 | 1 | 0.9865 | 1 |
| A0A1U8IPT1 | 1 | 0.3600 | 0 | 0.9687 | 1 |
| A0A3Q7EKL1 | 1 | 0.9200 | 1 | 0.9663 | 1 |
| B7F9I5 | 1 | 0.1800 | 0 | 0.2117 | 0 |
| Q6F368 | 1 | 0.0900 | 0 | 0.2899 | 0 |
| Q8L7T9 | 1 | 0.0900 | 0 | 0.9795 | 1 |

#

# Section S2: Evaluation of Our Methodology Multiclass DNA/RNA/SSB Dataset

To further assess the effectiveness of our method, we applied it to a multiclass DNA-binding protein dataset recently analyzed by Wu and Guo (2024). This dataset consists of four classes: RNA-binding proteins (RBP), single-stranded DNA-binding proteins (SSB), double-stranded DNA-binding proteins (DSB), and non-nucleic acid-binding proteins (NABP). We followed their dataset split for a fair comparison: 81% training, 9% validation, and 10% testing. When evaluated on the test set, our ProtT5-based, SaProt-based, and combined models achieved better accuracies significantly outperforming Wu and Guo's results. Similar trends were observed in the validation set, underscoring the robustness of our approach.

Table: Comparison of accuracies in multi-class DNA-binding dataset with ProtT5, SaProt-based, and combined models

| **Tool** | **RBP** | **DSB** | **SSB** |
| --- | --- | --- | --- |
| Wu and Guo ^1^ | 0.7030 | 0.7960 | 0.3940 |
| PLM-DBPs (ProtT5 Based) | 0.9146 | 0.9335 | **0.8571** |
| PLM-DBPs (SaProt Based) | 0.8930 | 0.8980 | 0.8080 |
| PLM-DBPs (Final Model) | **0.9252** | **0.9475** | 0.8566 |

1. Siwen Wu, Jun-tao Guo, Improved prediction of DNA and RNA binding proteins with deep learning models, Briefings in Bioinformatics, Volume 25, Issue 4, July 2024, bbae285, https://doi.org/10.1093/bib/bbae285

**Figure S1:** *2D UMAP features visualization of the penultimate layer features, showing well-separated clusters for RBP (Green), DSB (Blue), SSB (Red), and NABP (Orange) classes, demonstrating the ProtT5-based ANN model's effectiveness in multiclass classification.*
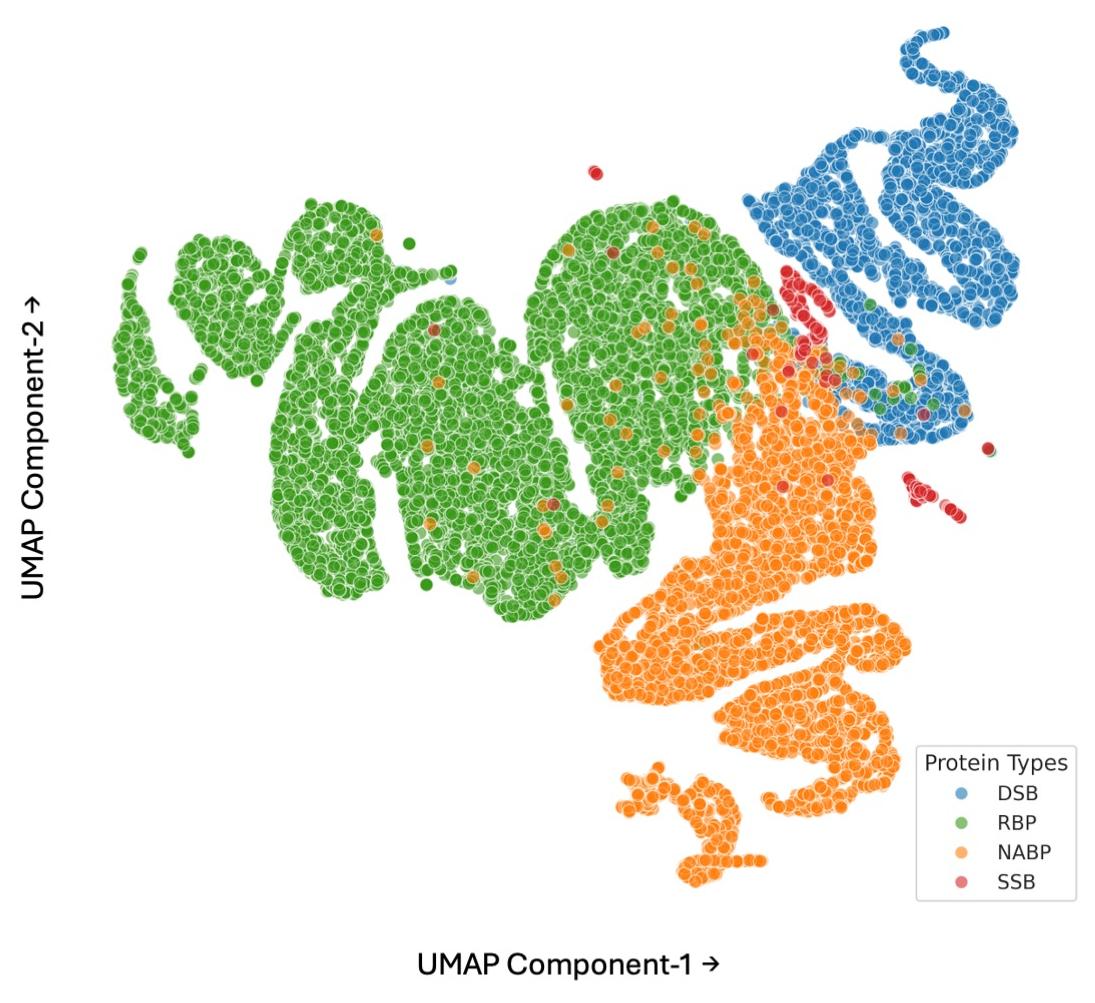


Figure S1 shows a 2D UMAP visualization of features extracted from the penultimate layer of our model, clearly illustrating well-separated clusters for each class: RBP (Green), DSB (Blue), SSB (Red), and NABP (Orange). Overall, these results demonstrate that the PLM-based feature is not only effective for plant-based DNA-binding prediction but also generalizes well to broader DNA- and RNA-binding tasks involving multiple classes.

#

# Section S3: Training statistics of the ProtT5 and SaProt-based ANN Models

We can observe a smooth decrease in loss and a consistent increase in accuracy, and close alignment between the training and validation accuracy/loss indicates that the model has learned effectively.


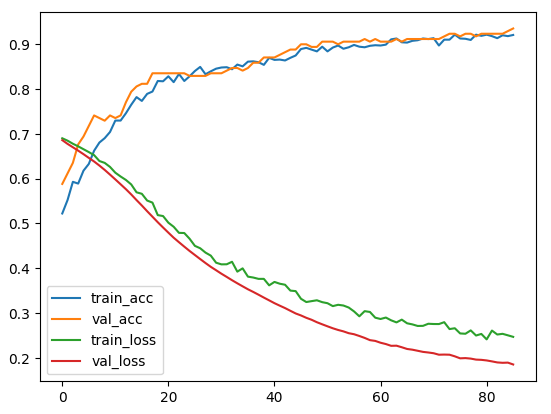


**Figure-S2.** *Training statistics of the ProtT5-based ANN model presenting accuracy and loss curve for training and validation.*


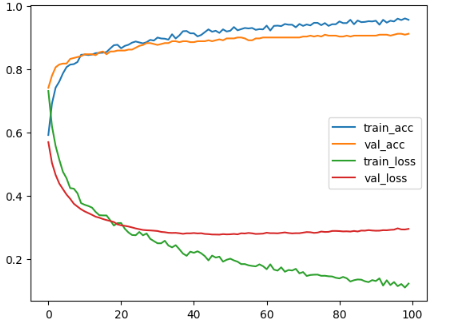

**Figure S3.** *Training statistics of the SaProt-based ANN model presenting accuracy and loss curve for training and validation.*

# Section S4: Proteins in Our Test Sets that Overlapped with ESM-DBP’s Training Set

Q6R0H1, Q84WU6, Q7XHR2, Q9LV52, O24621, Q8L7L8, Q9FHM5, F4IED2, F4JN35, Q00958, Q93WJ9, Q9SFD8, O22264, O49782, Q6R053, Q9FJV5, Q9LQX5, Q9LVW4, Q9M0K4, Q9SAK5, Q9FIM4, Q9LX82, Q8VY64, Q94BZ5, Q9LUH8, Q9M0F8, Q9S7U5, O04336, Q1PDN3, Q8S3Q9, Q42575, Q93ZH2, Q941I2, Q9FX84, Q6Z528, Q5Z5I4, Q6H7J5, Q6IEN1, Q32SG4, Q9C9F0, Q9LTA2
